# Ketamine affects the integration of developmentally generated granule neurons in the adult stage

**DOI:** 10.1186/s12868-019-0542-4

**Published:** 2019-12-18

**Authors:** Zhanqiang Zhao, Bing Li, Yuqing Wu, Xujun Chen, Yan Guo, Yang Shen, He Huang

**Affiliations:** 1 Department of Anesthesiology, Jiangning Hospital of Traditional Chinese Medicine, Nanjing, China; 2 Jiangsu Province Key Laboratory of Anesthesiology, Xuzhou, China; 3 Department of Anesthesiology, First Affiliated Hospital With Nanjing Medical University, Guangzhou Road 300, Nanjing, 210029 Jiangsu People’s Republic of China

**Keywords:** Ketamine, Dentate gyrus, Functional integration, Neural circuits, Morris water maze, Learning, Rat

## Abstract

**Background:**

Ketamine has been reported to cause neonatal neurotoxicity in a variety of developing animal models. Various studies have been conducted to study the mechanism of neurotoxicity for general anesthetic use during the neonatal period. Previous experiments have suggested that developmentally generated granule neurons in the hippocampus dentate gyrus (DG) supported hippocampus-dependent memory. Therefore, this study aimed to investigate whether ketamine affects the functional integration of developmentally generated granule neurons in the DG. For this purpose,the postnatal day 7 (PND-7) Sprague-Dawley (SD) rats were divided into the control group and the ketamine group (rats who received 4 injections of 40 mg/kg ketamine at 1 h intervals). To label dividing cells, BrdU was administered for three consecutive days after the ketamine exposure; NeuN+/BrdU+cells were observed by using immunofluorescence. To evaluate the developmentally generated granule neurons that support hippocampus-dependent memory, spatial reference memory was tested by using Morris Water Maze at 3 months old, after which the immunofluorescence was used to detect c-Fos expression in the NeuN^+^/BrdU^+^ cells. The expression of caspase-3 was measured by western blot to detect the apoptosis in the hippocampal DG.

**Results:**

The present results showed that the neonatal ketamine exposure did not influence the survival rate of developmentally generated granule neurons at 2 and 3 months old, but ketamine interfered with the integration of these neurons into the hippocampal DG neural circuits and caused a deficit in hippocampal-dependent spatial reference memory tasks.

**Conclusions:**

In summary, these findings may promote more studies to investigate the neurotoxicity of ketamine in the developing brain.

## Background

Every year, millions of children are exposed to a variety of surgeries. Ketamine, an N-methyl-d-aspartate (NMDA) receptor antagonist, is widely used for sedation in a clinical setting for analgesia in children who are undergoing painful procedures [1–3]. However, recent results have demonstrated that ketamine could increase potential risks for brain development. For instance, ketamine that was administered to developing rats resulted in a significant level of neurotoxicity [4]. The intravenous administration of ketamine during the first week of life caused long-lasting cognitive deficits in rhesus monkeys [5]. In vitro experimental evidence has also shown that ketamine could cause apoptosis of neurons that were derived from human embryonic stem cells [6]. Given these data, the safe use of ketamine for pediatric anesthesia has been the subject of concern for both anesthesiologists and the public. The mechanisms by which ketamine induces neurotoxicity in the developing brain remain to be determined.

The brain growth spurt (BGS) lasts from the end of pregnancy to the first 2–3 weeks after birth in rodents, and the corresponding BGS period in humans begins in the last trimester of pregnancy and continues until 2 years after birth [7]. During this period, developmentally generated hippocampal granule neurons normally migrate, survive in the granule cell layer (GCL) and send out axons to establish synaptic connections with pyramidal cells in hippocampal CA3, and functionally incorporate into pre-existing neural circuits (granule neurons-CA3-CA1) [8, 9]. Our previous study had suggested that neonatal ketamine exposure could interfere with the postnatal neurogenesis of the hippocampal dentate gyrus (DG), including the inhibition of neural stem cell (NSC) proliferation and astrocytic differentiation, the promotion of neuronal differentiation, the inhibition of astrocytic growth and the neuronal migration in GCL [10].

Previous studies have demonstrated that granule neurons that are generated in the early postnatal days have numerically dominated the adult hippocampal DG [11] and the new generated neurons have played important roles in the formation of hippocampal-dependent spatial learning and memory function [12, 13]. In addition, researchers found that the expression of immediate early genes (IEGs, such as c-Fos) were regulated by neuronal activity (such as memory testing), and immunofluorescence approaches have made it possible to estimate the proportion of developmentally generated granule neurons that have been functionally integrated into hippocampal memory networks by calculating the likelihood of IEG expression in NeuN^+^/BrdU^+^ cells [13, 14]. Given these previous findings, the purpose of this experiment was to investigate the effect of ketamine on the fates of developmentally generated granule neurons during the adult stage by using the in vivo neonatal ketamine exposure model.

## Materials and methods

### Animal treatment

All animal experiments were carried out according to the Guide for the Care and Use of Laboratory Animals of the National Institutes of Health (Publication No. 85-23, revised 1985). The experiments were approved by the Institutional Animal Care and Use Committee of the Nanjing Medical University (No: 15030254). Sprague–Dawley (SD) dams with pups were bred in our colony in a temperature-controlled (22–23 °C) room on a 12 h light/dark cycle (lights on at 8:00 a.m.) with free access to food and water. Fourty postnatal day 7 (PND-7) SD male rat pups (11–14 g) were used in our experiment and were randomly assigned to ketamine-treated and sham-treated groups. The grouping method was performed by using the methods described in our previous study [10]. In the anesthesia group, ketamine was diluted in 0.9% normal saline, and PND-7 rats were intraperitoneally administered with 40 mg/kg doses of ketamine in four injections at 1 h intervals (40 mg/kg × 4 injections). The ketamine injection program was explored through the preliminary experiment. In the sham-treated group, rats received an equal volume of 0.9% normal saline. Temperature probes were used to facilitate the control of temperature at 36.5 ± 1 °C by using computer-controlled heater/cooler plates that were integrated into the floor of the chamber. Between each injection, animals were returned to their individual chambers to help in maintaining body temperature and to reduce stress. We found that the movement and the righting reflex were disappeared in all animals after 40 mg/kg ketamine injection, and animals were completely unresponsive during the 1 h intervals between injections. These findings suggested that four injections of 40 mg/kg ketamine with 1 h intervals could exert the satisfactory anesthesia effect and all animals could survive after the anesthesia.

### Morris Water Maze test (MWM)

The apparatus and behavioral procedures of the MWM test have been previously described [10]. Behavioral testing was conducted in a circular, black painted pool (180 cm diameter, 50 cm deep). The water temperature was maintained at 25 ± 1 °C. An invisible platform (10 cm diameter) was submerged 1 cm below the water surface and was placed in the quadrant III, which was determined by using 4 starting locations (defined as I, II, III and IV). There was a 90 ° angular offset between each pair of starting locations. During 5 consecutive training days, the experiments were conducted in a dimly lit and quiet laboratory setting, we placed a lamp in a corner of the laboratory and kept the same light level used for both training and testing period, all animals could detect extra maze visual cues and learn how to locate the platform. The rats were trained four times per day, with the different starting position being randomized for each rat. When the rat found the platform, it was allowed to stay on the platform for 30 s. If a rat did not find the platform within 120 s, the rat would be gently guided to the location and allowed to stay on the platform for 30 s, and the latency time in finding the hidden platform was recorded as 120 s. The average escape latency from the four trials was represented as the daily result for each of the rats. For a given rat, the starting position was different across all 5 days of training. Following the completion of the training, spatial memory was assessed in the probe tests, in which the hidden platform was removed. The animals were placed in the quadrant opposite to the quadrant that had contained the platform and allowed to swim freely for 120 s. The paths of each of the animals were tracked by using a computerized video system. The number of times that the entire body of a rat crossed the previous platform area were recorded. The total swim distance and the average speed of each animal during the probe test were also need to be analyzed. After every trial, each rat was placed on a heater plate for 1 to 2 min until they were dry, after which they were returned to their chambers. The data were analyzed by using software for the MWM (Jiangsu Province Key Laboratory of Anesthesiology, Xuzhou Medical University, Xuzhou, China).

### Experimental design

Experiment 1 evaluated the survival rate of developmentally generated granule neurons in the hippocampal DG during adult stage. The PND-7 rats received three consecutive BrdU (5-bromo-2-deoxyuridine; Sigma) injections intraperitoneally, at a dosage of 100 mg/kg, on PND-7, 8 and 9 after administered with normal saline or ketamine, then 2 groups of rats were weaned at PND-35, after which they were housed in cages with free access to food and water for up to 2 months (five animals per group) (Fig. 3a). Then, all animals were deeply anesthetized with 40 mg/kg ketamine at 2 months old and transcardially perfused with 0.9% normal saline, followed by a transfusion with 4% paraformaldehyde. To visualize the dividing neurons in the early postnatal DG, the NeuN^+^/BrdU^+^ cells in the hippocampal DG were examined by using double-immunofluorescence staining (5 tissue sections per group).

**Fig. 1:**
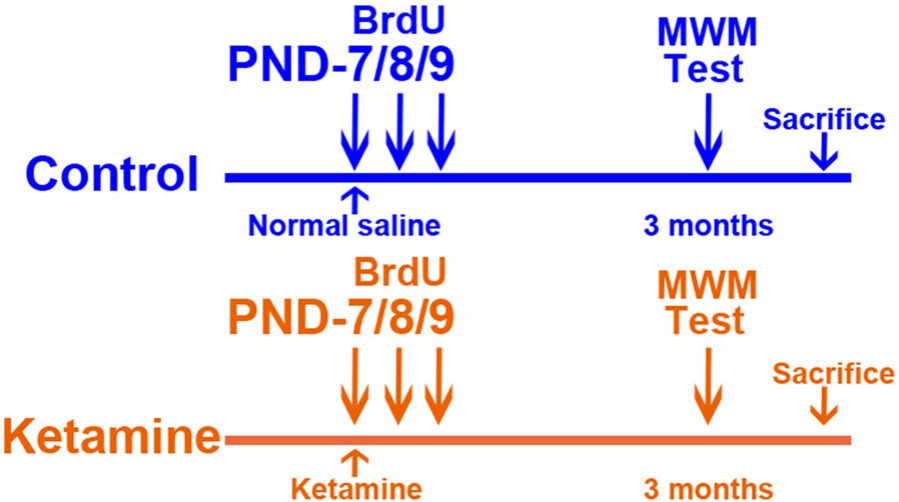
Experimental protocol for the administration of ketamine in test rats

Experiment 2 evaluated the integration rate of developmentally generated granule neurons into the hippocampus-dependent memory networks in the DG (Fig. 1). The PND-7 rats received three consecutive BrdU injections intraperitoneally on PND-7, 8 and 9 after administered with normal saline or ketamine, then two groups of rats were weaned at PND-35, after which they were housed in cages with free access to food and water for up to 3 months old (six animals per group). Hippocampus-dependent memory was assessed following the training period in the MWM task. Then, all animals were deeply anesthetized with 40 mg/kg ketamine and transcardially perfused with 0.9% normal saline, followed by a transfusion with 4% paraformaldehyde. The previous study had suggested that the expression of c-Fos was regulated by the neural activity that occurs as an animal performs the hidden platform version of the water maze [13]. The c-Fos expression in NeuN^+^/BrdU^+^ cells was examined by triple-immunofluorescence staining. This approach was used to estimate whether developmentally generated granule neurons had been functionally integrated into hippocampal memory networks during adult stage. In this experiment, two groups of animals were sacrificed immediately after the completion of the MWM testing. The integration rate of developmentally generated granule neurons into the hippocampal memory networks was estimated by calculating the proportion of c-Fos^+^/NeuN^+^/BrdU^+^ cells in the hippocampal DG (5 tissue sections per group).

### Tissue preparation and immunofluorescence

The brains were postfixed in 4% paraformaldehyde and the coronal sections of the brains were cut consecutively at a thickness of 30 μm, at the point in which the hippocampus was initially exposed, the 15th section was taken and stored in PBS. The position of the hippocampus coronal sections selected in our study was approximately 2.80–2.85 mm posterior to the bregma for the 2 months old rats and approximately 2.90–2.95 mm posterior to the bregma for the 3 months old rats [15, 16].

For the NeuN/BrdU double-immunofluorescence staining, the BrdU antigen was exposed by incubating the sections in 2-normal hydrochloric acid for 30 min at 37 °C, then the sections were washed by PBS. The blocking of nonspecific epitopes with 10% donkey serum in PBS (which contained 0.3% Triton-X) for 2 h at room temperature preceded an overnight incubation at 4 °C with the primary antibodies against NeuN (Mouse anti-NeuN monoclonal antibody; 1:200; Millipore, Massachusetts, USA) and BrdU (Rabbit anti-BrdU monoclonal antibody; 1:500; Abcam, San Francisco, USA). On the next day, the sections were incubated with the appropriate secondary fluorescent antibodies (Invitrogen Carlsbad, USA) for 2 h at room temperature.

For the Fos/NeuN/BrdU triple labeling, identical procedures were performed by using a primary rabbit anti-c-Fos polyclonal antibody (1:200; Abcam), a mouse anti-NeuN antibody (1:200; Millipore) and a rat anti-BrdU monoclonal antibody (1:500; Abcam). On the next day, the sections were incubated with the appropriate secondary fluorescent antibodies (Invitrogen) for 2 h at room temperature.

### Imaging

The single-plane images of the stained sections were taken by using a laser scanning confocal microscope (Fluoview 1000, Olympus, Japan), and a skilled pathologist, who was blinded to the study conditions, examined the labeled sections and portrayed the scale of hippocampal DG in the brain slice in the fluorescence image. The numbers of double-positive or triple-positive cells in the hippocampal DG were manually quantified by using Image-Pro Plus software (Media Cybernetics Inc., Bethesda, USA).

### Brain tissue harvest and western blot analysis

In order to observe whether neonatal ketamine exposure induces neural apoptosis in the hippocampal DG at 2 months old and 3 months old, levels of caspase-3 expression were measured at these time points. Rats in the sham group and ketamine group were deeply anesthetized with ketamine and decapitated at 2 months old (three animals per group) or 3 months old (three animals per group). The hippocampal DG tissue was dissected carefully with an stereo microscope (Leica EZ4HD). The harvested hippocampal DG tissues were homogenized on ice using lysate buffer plus protease inhibitors. The lysates were centrifuged at 14,000 rpm for 15 min at 4 °C and were resolved by 12% polyacrylamide gel electrophoresis, and the target proteins were transferred to nitrocellulose membranes. The blots were incubated with blocking buffer for 2 h at room temperature and then incubated for 24 h at 4 °C with the primary antibodies rabbit anti-caspase-3 antibody (1:1000 dilution; Cell Signaling Technology) and β-tubulin (1:10,000 dilution; Abcam). The membranes were then incubated with the appropriate secondary alkaline phosphatase-conjugated antibody (Abcam, dilution factors included Tris–Hcl, NaCl, tween20) for 1 h at room temperature. The band intensity was quantified using Image J software. We quantified the Western blots in two steps. First, we used β-tubulin levels to normalize (e.g., determining the ratio of caspase-3 to β-tubulin amount) protein levels. Second, we presented changes in ratio levels in rats undergoing ketamine anesthesia as a percentage of those in the control group. One hundred percent of ratio level changes refer to control group for the purpose of comparison with experimental conditions.

### Statistical analysis

The statistical analysis was conducted by using SPSS 13.0 (SPSS Inc., Chicago, USA), and the graphs were created by using GraphPad Prism 5 (GraphPad Software Inc., La Jolla, USA). The data were analyzed by using the Mann–Whitney U test. The interaction between the time and group factors, which was determined by using a two-way ANOVA, was used to analyze the differences in escape latency between the rats in the control group and the rats that were treated with ketamine in the MWM. The data are presented as the mean ± SD, and *P *< 0.05 was considered to be statistically significant.

## Results

### Exposing ketamine to PND-7 rats caused spatial memory impairment at 3 months old in the MWM test

**Fig. 2:**
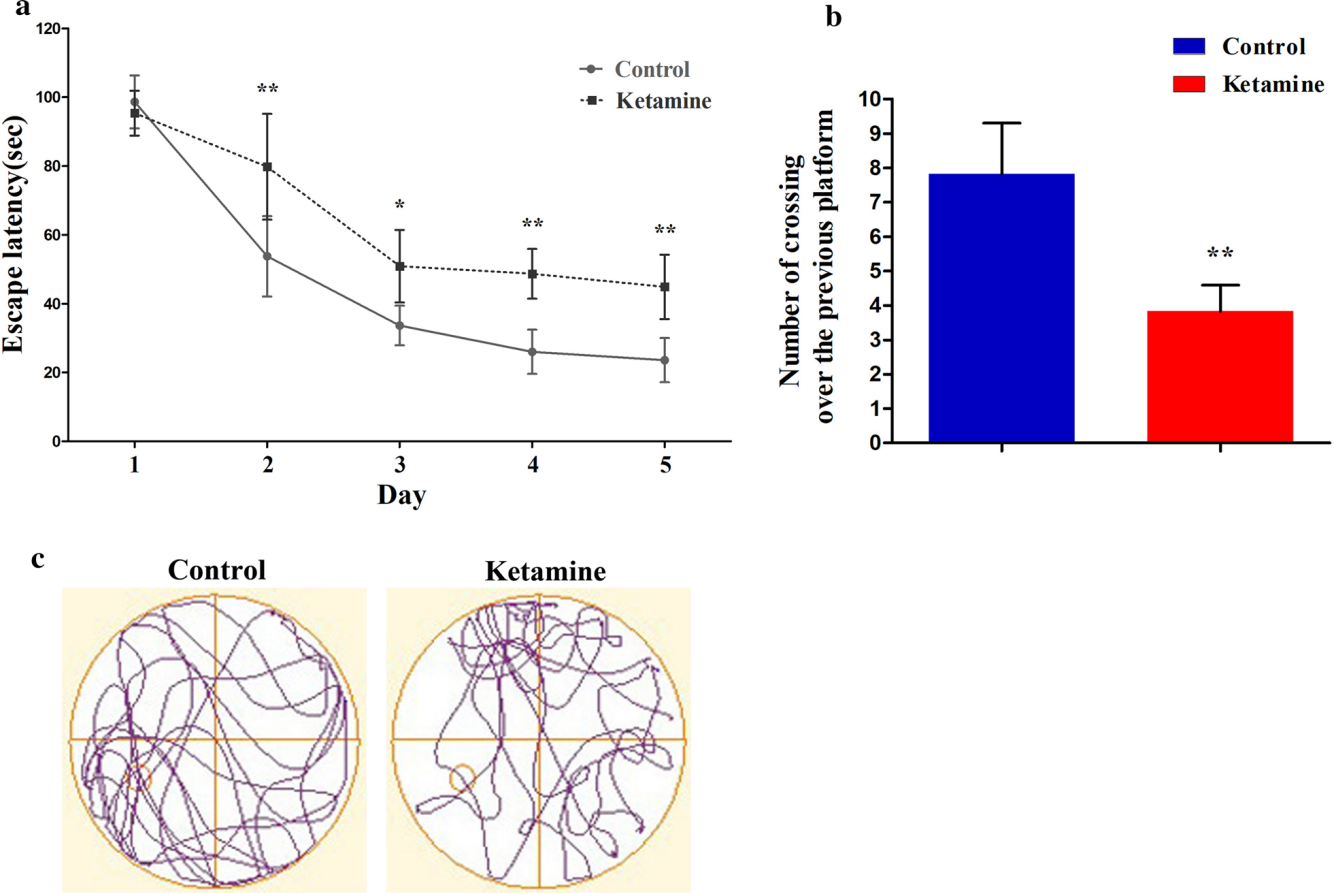
Anesthesia with ketamine (40 mg/kg × 4 injections) in neonatal rats at postnatal day 7 (PND-7) induces learning and memory impairment in the adult stage. Compared with the control group, ketamine anesthesia significantly increased the latency times of rats swimming in the Morris Water Maze (MWM) (**a**). In the memory retrieval tests, the time that each rat spent in the target quadrant within 120 s was significantly reduced in the ketamine group compared to that of the control group (**b**). The numbers of crossovers of the previous platform site within 120 s was significantly reduced in the ketamine group compared to that of the control group (**c**). Typical path charts of space exploration were exhibited (**d**). Data are presented as the mean ± SD, six animals per group. ^*^P < 0.05, ^**^P < 0.01 vs. the control group

Comparison of the time that each rat spent in reaching a platform during the training phase (the escape latency), the latency to find the hidden platform in the 2 groups of rats had a reduced time as the training progressed. However, in both the sham group and ketamine group, rats displayed reduced escape latencies as training progressed (Fig. 2a). In the memory retrieval tests, the numbers of crossovers of the previous platform site (ketamine: 4 ± 0.75 vs. sham: 8 ± 1.47, *P *< 0.01; Fig. 2b) within 120 s were significantly reduced in the ketamine group compared to that of the sham group. The typical track charts in the memory retrieval test are shown in Fig. 2c. We analyzed the tracking data, there were no significant difference in the total traveled distance of each rat between control and ketamine group (sham: 3174 ± 129.9 cm vs. ketamine: 3151 ± 114.4 cm, *P *= 0.74). In addition, there were no significant difference in the average speed of each animal between control and ketamine group (sham: 28.45 ± 1.19 cm/s vs. ketamine: 28.69 ± 0.94 cm/s, *P *= 0.74). These analyses suggest that the performance deficits observed in the ketamine group cannot be accounted for by simple locomotion impairments. Taken together, these findings suggested that exposing ketamine (40 mg/kg × 4 injections) to PND-7 rats could cause hippocampal-dependent learning and memory impairments in the adult stage.

### Exposing ketamine to PND-7 rats did not affect the survival rate of developmentally generated granule neurons by using immunofluorescence staining and western blot

**Fig. 3:**
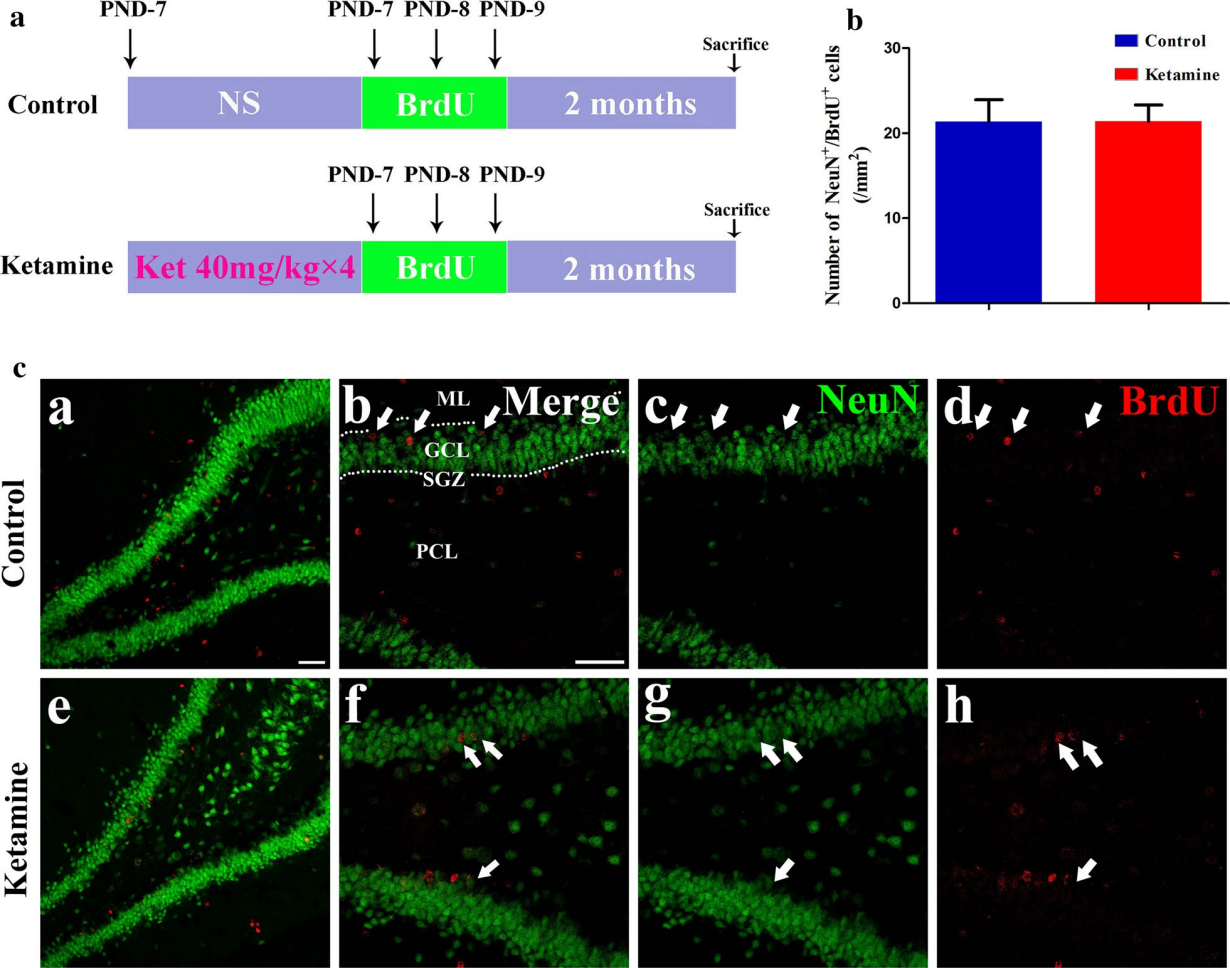
Neonatal ketamine (40 mg/kg × 4 injections) exposure did not affect the survival rate of developmentally generated neurons in the hippocampal DG during the adult stage. Experimental protocol (**a**). The density of developmentally generated neurons in the hippocampal DG coronal sections was not different between the sham and ketamine groups at 2 months. The Y axis “(/um^2^)” represents the density of NeuN^+^/BrdU^+^ cells in the DG (**b**). The developmentally generated granule neurons in the hippocampal DG were labeled with primary antibodies against NeuN (Green) and BrdU (Red). The representative images were acquired by using a laser scanning confocal microscope (**c**; magnification: a and e  ×  20, b–d and f–h  ×  40); the scale bar was 50 μm (**a** and **b**). The filled arrows point to the NeuN/BrdU double-labeled cells. Data are presented as the mean ± SD, 5 tissue sections per group. *NS*  normal saline, *GCL* granule cell layer, *SGZ*  subgranular zone, *ML* molecular layer, *PL* polymorphic cell layer

**Fig. 4:**
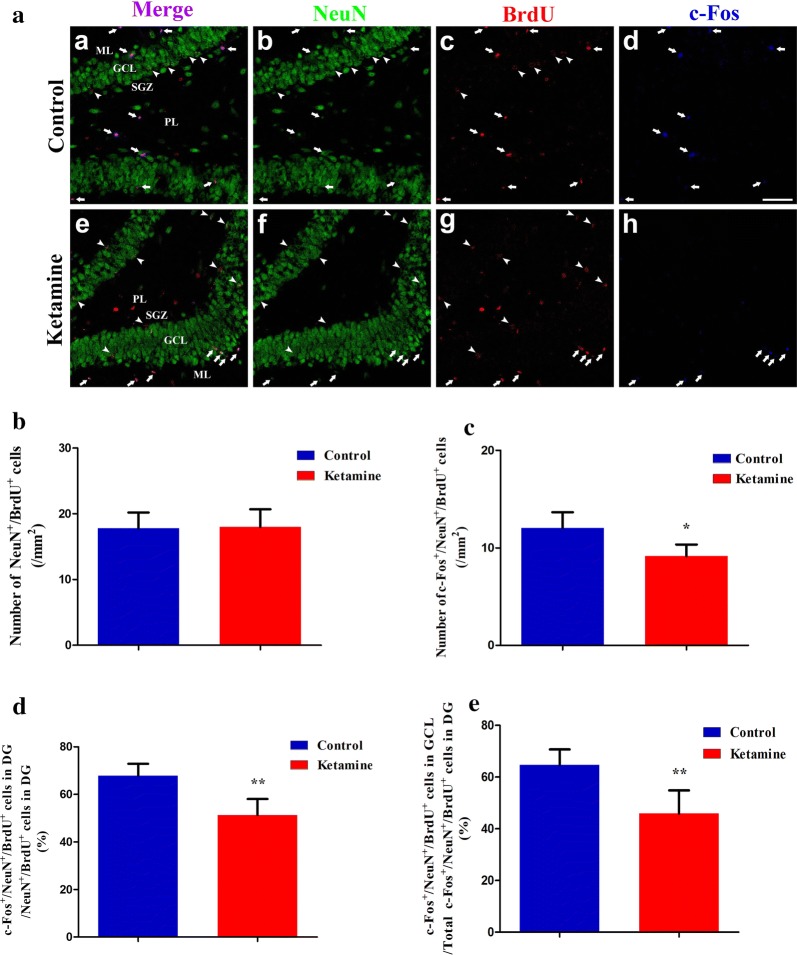
Neonatal ketamine exposure interfered with the functional integration of developmentally generated neurons into the hippocampal DG circuits during the adult stage. High magnification examples of c-Fos (blue), NeuN (Green) and BrdU (Red) immunofluorescences in the DG were captured by using a laser scanning confocal microscope, following the Morris Water Maze testing (**a**; magnification: a–h  × 40); the scale bar was 50 μm. The arrowheads point to the NeuN/BrdU double-positive cells. The filled arrows point to the c-Fos/NeuN/BrdU triple-positive cells. Ketamine could significantly decrease the density of triple-positive cells in the hippocampal DG (**c**), and the percentage of double labeled cells that were also triple labeled was reduced in the ketamine group (**d**). The percentage of NeuN/BrdU-positive cells that expressed c-Fos in the GCL was decreased in the ketamine group (**e**). Data are presented as the mean ± SD, 5 tissue sections per group. ^*^P < 0.05, ^**^P < 0.01 vs the control group. *GCL* granule cell layer, *SGZ* subgranular zone, *ML* molecular layer, *PCL* polymorphic cell layer

To investigate the effect of ketamine on the survival rate of developmentally generated neurons in the hippocampal DG, we used BrdU labeling and tracked the fates of developmentally generated granule neurons during the adult stage via double-immunofluorescence staining. NeuN and BrdU colabeled cells were defined as developmentally generated neurons that were generated at the time of BrdU injections. The experimental protocol is shown in Figs. 1 and 3a. According to our findings, the densities of developmentally generated neurons in the representative hippocampal DG coronal sections were not different between the sham and ketamine groups at 2 months (21 ± 2.56/mm^2^ vs 21 ± 1.91/mm^2^, *P *= 0.989; Fig. 3b) and at 3 months (18 ± 2.42/mm^2^ vs 18 ± 2.70/mm^2^, *P *= 0.907; Fig. 4b).

**Fig. 5:**
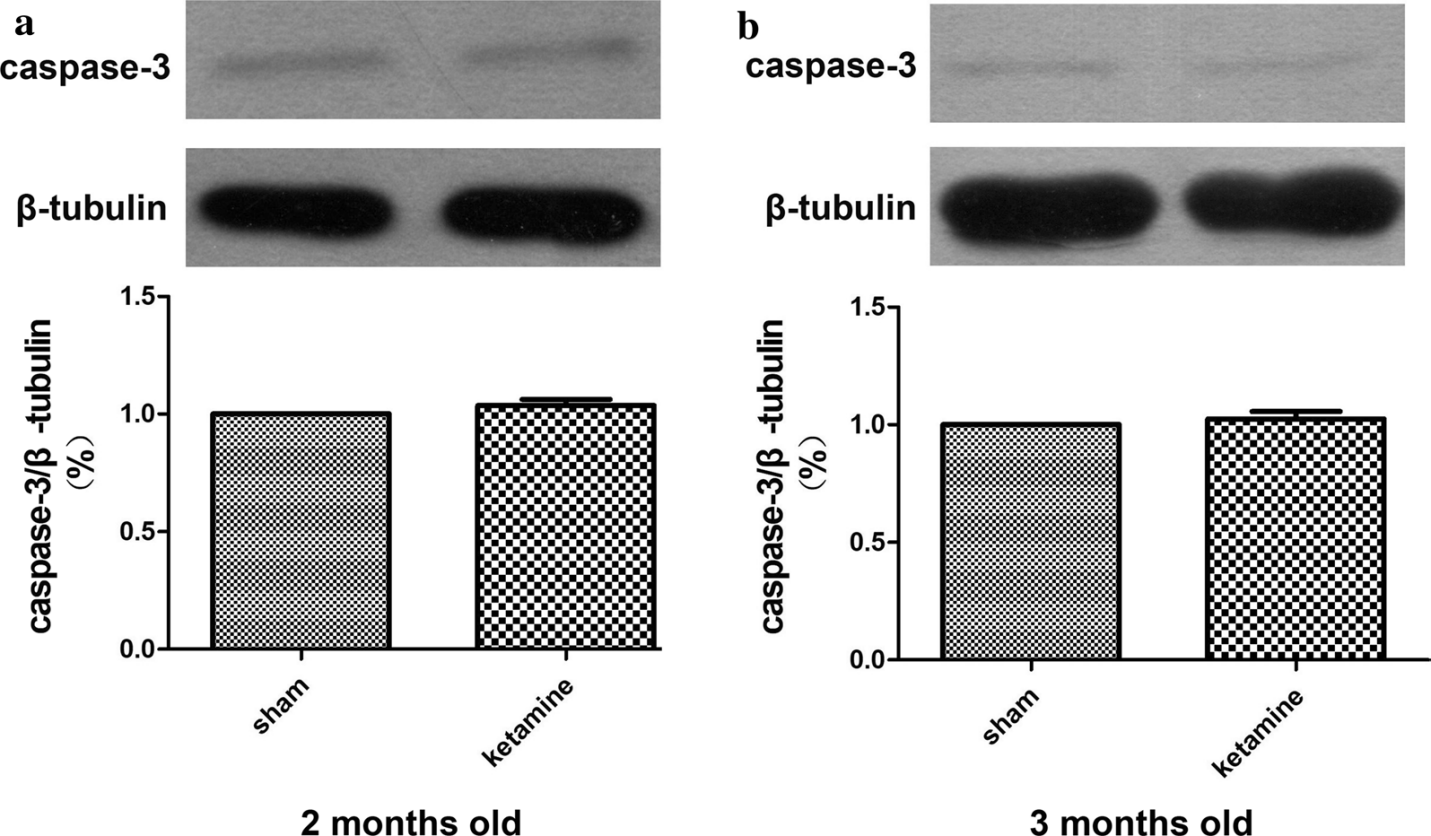
Neonatal ketamine (40 mg/kg × 4 injections) exposure did not affect the expression of caspase-3 in the hippocampal DG tissues of rats at 2 months old (**a**) and 3 months old (**b**) on western blot analysis. There was no statistically significant difference in the amounts of caspase-3 in the rat brain tissues following the ketamine anesthesia or control condition at 2 months old (**a**) and 3 months old (**b**). Data are presented as the mean ± SD, three animals per group

Next, we detected the expression of caspase-3 in the hippocampal DG by western blot. The result showed that four injections of 40 mg/kg ketamine with 1 h intervals did not significantly affect the expression of caspase-3 in the hippocampal DG at 2 months (*P *= 0.104, Fig. 5a) and 3 months old (*P *= 0.161, Fig. 5b). These results suggested that 40 mg/kg ketamine × 4 injections in PND-7 rats did not affect the survival rate of developmentally generated neurons in the hippocampal DG in the adult stage.

### Neonatal ketamine exposure interfered with the functional integration of developmentally generated neurons in the adult stage

The expression of c-Fos has been used as a neuronal activity marker in previous experiments [13]. To investigate the effect of ketamine on the integration of developmentally generated neurons in the hippocampal DG circuits, we observed the c-Fos/NeuN/BrdU triple-positive cells and the NeuN/BrdU double-positive cells by using immunofluorescence staining following the completion of the Morris Water Maze tests. The experiment protocol is shown in Fig. 1. We observed that ketamine could significantly decrease the density of c-Fos/NeuN/BrdU triple-positive cells in the hippocampal DG compared to that in the sham group (ketamine: 9 ± 1.19/mm^2^ vs sham: 12 ± 1.63/mm^2^, *P *= 0.013; Fig. 4c) and the percentage of double labeled cells that were also triple labeled in the ketamine group were also reduced (ketamine: 51 ± 6.74% vs sham: 68 ± 5.07%, *P *= 0.0024; Fig. 4d). The cell layer in the hippocampal DG mainly includes GCL, polymorphic cell layer (PCL) and molecular layer (ML). Only the normal migration of granule neurons to the GCL can exert normal function, abnormal migration of granule neurons in the GCL is associated with hippocampal-specific cognitive deficits [17]. According to our findings, the ratio of triple-positive cells in the GCL to total triple-positive cells in the DG (ketamine: 46 ± 8.99% vs sham: 65 ± 6.03%, *P *= 0.0046; Fig. 4e) was decreased in the ketamine group, compared to the sham group.

The present results suggested that 4 treatments with 40 mg/kg ketamine in PND-7 rats interfered with the normal localization of developmentally generated neurons in the hippocampal DG, it may be caused by suppressing the number of astrocytes in the hippocampal DG after neonatal ketamine exposure [10], because astrocytes played a support role in the normal migration of developmentally generated neurons [18]. In addition, we found that ketamine interfered with the functional integration of neurons into the hippocampal-dependent spatial memory circuits. The above factors may be associated with learning and memory impairment in the MWM test induced by neonatal ketamine exposure.

## Discussion

Ketamine is one of the most commonly used general anesthetics, particularly in the field of pediatric anesthesia. The growing data suggest that, during the period of brain growth spurts, ketamine can induce widespread neuronal apoptosis in parallel with long-term memory and learning abnormalities [4, 5]. According to these results, the safe use of ketamine in surgical and intervention procedures has become a major health issue of interest to the public [19, 20]. Therefore, it is necessary to clarify the mechanism of the neurotoxicity of ketamine in the developing brain.

Neurogenesis in the hippocampal DG plays a crucial role in the formation of the structure and function of the hippocampus [21, 22]. In rodent animals, the granule neurons are continuously generated in the subgranular zone of the hippocampal DG from the 14th day of gestation until the adult stage, and approximately 80% of the granule neurons in the DG are produced postnatally, with a peak at approximately 7 days after birth [23]. Muramatsu et al. found that postnatally generated granule neurons can numerically dominate the adult hippocampal DG [11]. However, the total number of granule neurons may not be as important for hippocampal function as the efficient integration of these neurons into the neural circuits [21, 24]. The key point for estimating how these developmentally generated granule neurons contribute to hippocampal-dependent spatial memory is to explore whether they can migrate into the normal positions of the GCL and whether they can incorporate into neural circuits, in order to meet the functional demand. Normally, newly generated granule cells migrate into the GCL, send their axons to CA3 field [25–27], and mature into functional neurons that are incorporated into the hippocampal neural circuit (granule cells–CA3–CA1 loop) [8]. These new generated neurons are thought to play a significant role in hippocampal synaptic plasticity [28].

BrdU is a classically used tool for the detection of cell fates [29, 30]. In our experiment, neonatal rats received 100 mg/kg BrdU injections on PND-7, 8 and 9 after exposures to either saline or ketamine treatments [10]. We defined the NeuN/BrdU colabeled cells that were detected at 2 months and 3 months as the developmentally generated granule neurons that were generated at the time of BrdU injection by using a laser scanning confocal microscope. Our results suggest that the densities of NeuN/BrdU colabeled cells in the hippocampal DG at 2 months and 3 months were not different between the sham and ketamine groups, and ketamine did not significantly affect the expression of caspase-3 in the hippocampal DG at 2 months and 3 months old. Collectively, these results demonstrate that the survival rate of developmentally generated granule neurons in the adult stage was not affected by neonatal ketamine exposure. However, in the MWM test at 3 months old, the latency of rats in locating the hidden platform in the ketamine group was significantly longer than that in the sham group. In the memory retrieval tests that followed the training period, the time spent in the target quadrant and the numbers of crossovers of the previous platform site within 120 s were significantly reduced in the ketamine group compared to those in the sham group. One key question is whether hippocampal-dependent learning and memory impairment in the adult stage was related to the abnormal integration of developmentally generated DG neurons that was induced by ketamine.

The IEG c-Fos expression has been used as a neuronal activity marker in previous experiments [13, 14]. The c-Fos expression is regulated by neural activity and therefore has been used to map neural activation, for example, learning and/or memory recall [14]. The c-Fos/NeuN/BrdU triple immunofluorescence labeling makes it possible to estimate the integration rates of developmentally generated granule neurons into the hippocampal-dependent memory networks [13]. In our experiment, BrdU and c-Fos immunofluorescence labeling were introduced to study the activation of developmentally generated granule neurons following memory recall. Our results demonstrated that spatial memory recall induced a relatively small proportion of c-Fos/NeuN/BrdU triple-positive cells in the hippocampal DG in the ketamine group, compared with the sham group. According to these findings, it was suggested that the abnormal integration of developmentally generated granule neurons into the hippocampal DG neural circuits may be an important determinant for long-term hippocampal-dependent cognitive deficits after neonatal ketamine exposure. A previous study reported that colchicine injection into the DG caused the impairment in hippocampal-dependent spatial memory, but the lesion was limited to the DG rather than other hippocampal regions [31]. This result suggested that the damage in the hippocampal DG alone could produce a hippocampal-dependent neurocognitive dysfunction.

Consistent with a previous study [17], our earlier findings indicated that the abnormal migration of developmentally generated granule neurons in the hippocampal DG was associated with hippocampal-dependent cognitive deficits [10]. Within our present study, it was demonstrated that ketamine could markedly decrease the proportion of triple-positive cells in the GCL, which was similar to the results from our previous study, which demonstrated that ketamine could markedly inhibit the migration of developmentally generated neurons and could affect their normal positions in the hippocampal DG [10].

The mechanisms by which ketamine (40 mg/kg × 4 injections) interfered with the functional integration of developmentally generated granule neurons in hippocampal DG circuits remain to be determined. A previous study suggested that the developing mitochondria were especially vulnerable to general anesthesia and it may be an important early target to study the anesthesia-induced developmental neurodegeneration [32]. We hypothesized that neonatal ketamine exposure may have disturbed the normal process of mitochondrial energy metabolism in the developmentally generated neurons instead of affecting their survival rate in the adult stage. Thus, our future studies will include the exploration of these potential mechanisms.

## Conclusions

In summary, our findings suggested that neonatal ketamine exposure may not affect the survival rate of developmentally generated granule neurons, but may interfere with their functional integration into the hippocampal circuits. These findings may account for the adult hippocampal-dependent neurocognitive dysfunction that is induced by neonatal ketamine exposure.

## Data Availability

The data that support the findings of this study are available from the corresponding author if needed.

## Abbreviations

DG: dentate gyrus
PND-7: postnatal day 7
NMDA: N-methyl-d-aspartate
BGS: brain growth spurt
NSC: neural stem cell
GCL: granule cell layer
PCL: polymorphic cell layer
MWM: Morris Water Maze test
